# Somatosensory restoration and neural control strategies in lower-limb prostheses

**DOI:** 10.1038/s44385-025-00050-w

**Published:** 2025-12-02

**Authors:** Dulce M. Mariscal, Brendan Driscoll, He Huang, Lee E. Fisher

**Affiliations:** 1 Rehab Neural Engineering Labs, University of Pittsburgh, Pittsburgh, PA USA; 2 Department of Physical Medicine & Rehabilitation, University of Pittsburgh, Pittsburgh, PA USA; 3 Department of Electrical and Computer Engineering, North Carolina State University, Raleigh, NC USA; 4 UNC/NC State Lampe Joint Department of Biomedical Engineering, North Carolina State University, Raleigh, NC USA; 5 UNC/NC State Lampe Joint Department of Biomedical Engineering, University of North Carolina at Chapel Hill, Chapel Hill, NC USA; 6 Department of Bioengineering, University of Pittsburgh, Pittsburgh, PA USA; 7 Center for Neural Basis of Cognition, Pittsburgh, PA USA; 8 Department of Biomedical Engineering, Carnegie Mellon University, Pittsburgh, PA USA

**Keywords:** Motor control, Brain-machine interface

## Abstract

People with lower-limb amputation cannot directly control or receive feedback from existing prostheses, but emerging technologies aim to address this gap. Some approaches focus on restoring somatosensation in the missing limb, while others record signals from residual muscles for prosthetic control. This review provides an overview of the current state of neuroprosthetics for somatosensory restoration and prosthetic control in lower-limb amputation, offering perspectives on integrating these technologies for bidirectional neuroprostheses.

## Introduction

Losing a lower limb can cause significant disability. It is estimated that approximately 150,000 people in the United States undergo lower extremity amputation every year^1^. For many people, using a prosthetic limb after amputation can facilitate ambulation, although currently available commercial prosthetic devices do not interact directly with the user’s nervous system^2^. This lack of interaction between the prosthetic limb and the amputee significantly reduces the sensory information the user receives and limits their control over the device.

Over the past two decades, much research effort has focused on restoring sensory feedback and improving neural control for upper-limb prosthetics. Technologies aimed at people with lower-limb amputation can potentially leverage this wealth of research and insights related to upper-limb prosthetics. However, there are significant differences to consider between these populations and between the functions of the upper and lower limbs. For instance, reaching involves aiming and voluntary movement control^3^, whereas walking relies heavily on spinal and cerebellar reflexes to coordinate gait dynamics^4^. Moreover, while a malfunction in an upper-limb prosthesis may cause the user to drop an object, improper functioning in a lower-limb prosthesis significantly increases the risk of falls. This means that the control strategies and requirements between these systems should differ^2,5^. Another important difference between upper- and lower-limb amputations is that approximately 82% of lower-limb amputations are due to vascular disease and diabetes^6^. These conditions can compromise sensory afferents in the residual limb, making it more difficult to evoke sensations that appear to originate from the missing limb^7^, reducing volitional control of muscles in the residual limb, and impairing wound healing^8^, a potential challenge for implantable technologies.

The majority of available lower-limb prostheses, both commercially-available and open source, are still passive devices that cannot be actively controlled. Powered lower-limb prostheses emerged two decades ago, and some became commercially available, such as the Power Knee developed by Össur (Iceland), Intuy Knee by Reboocon (China), Proprio Foot by Össur, and Empower Ankle by Ottobock (Germany), while other open-source limbs developed by research groups offer access to software for low-level controllers and hardware design for those with the technical skills to build a custom limb^8^. All these devices are operated with autonomous control to produce cyclic stepping patterns and joint power to assist various locomotive tasks^9^. However, the function of these modern prostheses is still limited, partly due to the lack of direct communication interfaces with the user. For example, autonomous control is often insufficient for non-cyclic tasks that depend on spontaneous feedforward neural control, such as anticipatory postural movements. Using neural signals to directly operate the prosthesis can potentially resolve this problem. Recent efforts have focused on developing neural interfacing systems that bridge the gap between the prosthesis user and the prosthetic limb. Some of these efforts have focused on restoring somatosensation from the missing foot and leg^10^, while others have attempted to develop neural control methods that allow amputees to control the prosthetic limb using neuromuscular signals from the residual muscles^11^.

The development of neural interfacing systems has focused on developing both invasive or non-invasive technologies. Invasive neuroprostheses require surgical procedures to modify body parts or implant electrodes that interface directly with the nervous system, enhancing prosthetic limb control and restoring sensory feedback. While invasive solutions can provide superior motor control and sensory integration, they are limited by surgical risks and cost barriers. On the other hand, non-invasive technologies are safer and more accessible options that offer alternative approaches; however, they often lack the fine control and intuitive sensory feedback that users need.

**Fig. 1:**
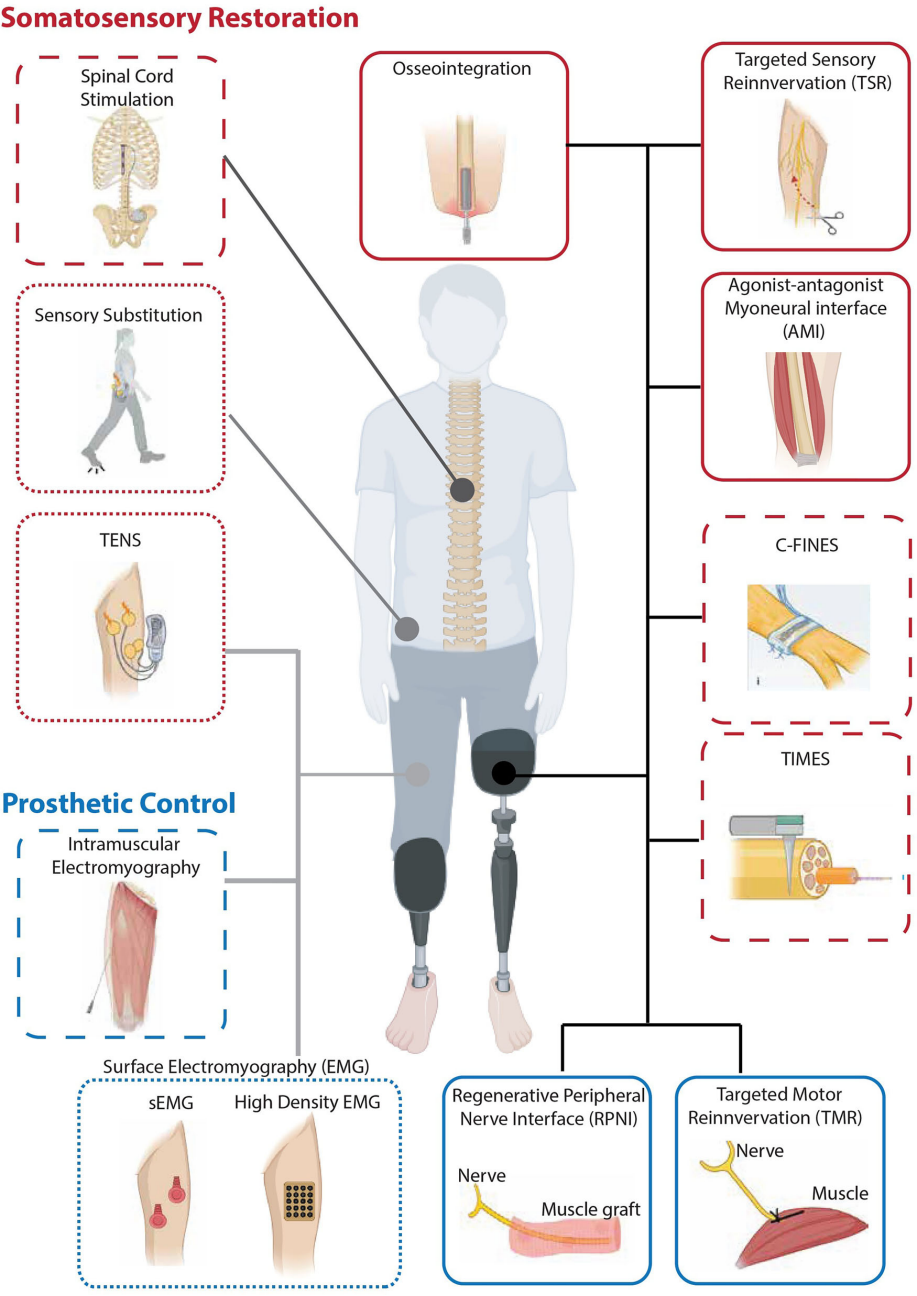
Overview of existing technologies for somatosensory restoration and prosthetic control in lower-limb amputees, as described in this review. The figure differentiates between two primary approaches: somatosensory restoration (red) and prosthetic control (blue). Technologies are organized into three categories: surgical procedures (solid lines), implantable technologies (dashed lines), and non-invasive technologies (dotted lines).The figure depicts two types of amputation: transtibial (left) and transfemoral (right), with lines indicating where each technology is applied. For instance, the black line denotes technologies used at the residual limb, while dark gray indicates spinal cord stimulation, implanted near participants’ spinal cords. Sensory substitution can occur anywhere on the body to replace the missing sensation; light gray signifies technologies used in the residual limb. Credit: C-FINE picture, reproduced with permission from ref. ^49^, under a Creative Commons license CC BY 4.0. Created with BioRender.com.

This review presents an overview of the current state of neuroprosthetics (invasive and non-invasive technologies) for somatosensory restoration and neural prosthetic control in lower-limb amputation (Fig. 1). It also aims to highlight some of the unknowns in the field that may limit our ability to create bidirectional lower-limb neuroprosthetics that integrate sensory feedback and prosthetic control to restore lower-limb amputees’ mobility.

## Somatosensory Restoration

Most of the efforts to restore somatosensory feedback after amputation have focused on the upper limb^12,13^. However, several studies in the past five years have focused on restoring somatosensory feedback from the amputated foot and leg^10,14^. Sensory input from the foot and leg is critical for walking and balance control^15^. People with lower-limb amputations rely on vision and the sensations from pressure applied by the socket on the residual limb to infer the state of the limb. This heavy reliance on vision and referred sensations on the residual limb requires additional cognitive focus to maintain stability and control their prosthesis, leading to an increased risk of falls^16^, asymmetric gait^17^, increased cognitive burden^18^, and reduced balance confidence^19^. Additionally, the mismatch between attempted and actual movements and the lack of sensory feedback from the prosthetic limb has been implicated as a potential cause of phantom limb pain (PLP)^20^.

The lack of sensory feedback from the prosthetic limb also impairs embodiment, a critical sensation of being integrated with the body and becoming part of the user’s anatomy^21,22^. Embodiment is a multifaceted experience in which the user has ownership (i.e., they feel as if it is their own) and agency (i.e., they feel as if they can control the movement) over the device and may enhance users’ willingness to adopt and maintain use of advanced prosthetic limbs. A crucial aspect of embodiment is the multisensory integration of sensory feedback and voluntary control. Studies have shown that restoring somatosensation enhances the ownership of the prosthetic device^23,24^ and their perception of its weight^25^. Furthermore, robust and intuitive control strategies have been found to enhance the ownership of the device^26^.

To restore somatosensory feedback in amputees, multiple research teams are developing somatosensory neuroprostheses that use electrical stimulation to evoke sensations that appear to emanate from the missing limb. Somatosensory neuroprostheses can be categorized as invasive (i.e., requiring surgical intervention to implant devices inside the body or modify anatomy in the residual limb) or non-invasive (i.e., devices placed on the skin’s surface). Invasive devices can achieve more intimate contact with the nervous system and a more stable long-term interface than non-invasive technologies, which must be frequently replaced on the skin. However, many people with amputation may not want to undergo surgical procedures and may prefer non-invasive technologies. Additionally, for people with poorly controlled diabetes (a common occurrence for people with lower-limb amputation), there may be an increased infection risk^27^, making them ineligible for elective surgical procedures. Below, we describe the primary invasive and non-invasive technologies used to restore somatosensory feedback in the lower limb and the functional improvements observed using these technologies.

### Invasive somatosensory neuroprostheses

Invasive neuroprostheses can be classified into two main categories: (1) surgical procedures that modify the anatomy of the residual limb and (2) implantable devices that deliver electrical stimulation to the patient’s nerves to evoke sensation in the missing limb.

#### Surgical procedures

*Agonist–antagonist Myoneural Interface (AMI)* is a surgical construct consisting of two muscle tendons (from a pair of antognist muscles) connected in series so that the contraction of one muscle stretches the other, evoking proprioceptive sensations from the stretched muscle^26^. AMI reconstructions have been performed in transtibial^26^ and transfemoral^28^ amputees. For transtibial amputations, typically two AMIs are used: one composed of the tibialis posterior and the peroneus longus responsible for ankle inversion and eversion movements, and a second, composed of the lateral gastrocnemius and the tibialis anterior responsible for ankle plantar flexion and dorsiflexion movements^26^. For transfemoral amputation, these muscles are no longer available, and instead an AMI associated with knee flexion and extension is created by connecting the rectus femorus with the biceps femorus^28^. This approach does not restore tactile feedback, but it has been shown that people with AMI surgeries have better control of prosthetic limbs^26^, improved walking speed, gait symmetry, and better kinetics while walking uphill, downhill, or up and down stairs compared with people with traditional amputation procedures^29^. Among the small cohort of people that have undergone this surgical approach at the time of amputation, they have not experienced post-amputation PLP^30,31^.

*Osseointegration* is a surgical procedure in which a titanium rod is inserted into the skeletal structure of the amputee’s residual limb. The rod is placed in the femur for transfemoral amputees, while, for transtibial amputees, the rod is placed in the tibia^32^. Differences in the diameter and length of the bones where the rod is placed (femur vs. tibia) can increase the risk of mechanical failure^33^. A prosthetic adaptor anchored to the bone protrudes out of the skin at the base of the limb^34^. Following the initial surgery, it may take patients up to 5 months before they are ready to walk on a prosthesis again^35^. Many benefits have been reported among patients who have successfully undergone these procedures, including improved proprioception and better performance on functional tests such as the 6-minute walking and timed up-and-go tests^35^. The direct interface with the skeletal structure allows patients to benefit from “osseo-perception,” in which they feel vibrations and forces transmitted through the bone in the residual limb via the osseo-implant, providing information about their prosthetic limb and how it interacts with the environment^34^. Osseo-perception enables individuals to distinguish between different vibration frequencies and perceive sounds at frequencies of 400 Hz or higher^36^. A major challenge for this technology is the high risk of infection from devices interfacing with the body’s skeletal structure through the skin. Although post-surgery infection rates have declined over time, studies still show rates between 18% and 63%^37^.

*Targeted Sensory Reinnervation (TSR)* is a surgical procedure that redirects the nerve that previously innervated the missing limb to an intact patch of skin somewhere else on the body^38^. The transferred nerve regrows, and connections between the skin and tactile sensory afferents are re-established. As a result, when pressure or vibration is applied to the remapped sensory area, the reinnervated skin creates a sensation that feels like it is coming from the missing limb^38,39^. Work from Gardetto (2021) has shown that in transtibial and transfemoral amputees, TSR can help to evoke sensation in the missing foot. Additionally, TSR can help to reduce the presence of PLP and improve scores on functional tests such as the timed up-and-go, four-step square, and 6-minute walking test^40^.

#### Implantable devices

There is a wide range of implantable technologies for restoring somatosensation, including devices to stimulate sensory fibers in peripheral nerves and the spinal cord. These technologies restore sensation by delivering trains of electrical stimulation pulses to activate sensory neurons in the residual nerve to evoke sensations that appear to emanate from the missing leg^41^. Sensors on the prosthetic limb modulate stimulation pulse trains so that the sensations appear to come from specific locations on the prosthesis.

#### Peripheral Nerve Stimulation

Peripheral nerve stimulation has been used to restore sensation in the upper and lower limbs^10,13^. This approach involves placing small electrodes intraneurally (inserted into the nerve) or epineurally (wrapped around the nerve without penetrating it) on the peripheral nerve in the residual limb (i.e., sciatic, tibial, or common peroneal nerves).

*Transversal Interfascicular Multichannel Electrodes (TIMEs)* are intraneural electrodes designed to penetrate the peripheral nerve transversely and are intended to selectively activate subsets of axons in different fascicles within the nerve^42^. TIMEs have been used to restore sensory feedback in upper^13^ and lower limbs^43^. In three people with lower-limb amputation, TIMEs have elicited sensations of touch, pressure, pulsation, proprioception, muscular contraction, temperature, tingling, and electricity in more than 20 positions over the phantom foot sole and lower limb^43,44^. Furthermore, in a small cohort of participants, sensory restoration via TIMEs reduced the metabolic cost of walking, cognitive efforts, and the presence of PLP^23,43^. Because intraneural electrodes disrupt the blood-nerve barrier, there is a substantial risk that glial scarring and other immunological responses could isolate the electrode from neurons and decrease their performance over time^45,46^. The long-term performance of these devices has not been characterized yet and will be an important step towards broader clinical translation.

*High-density Composite Flat Interface Nerve Electrodes (C-FINEs)* are epineural electrodes designed with patterned stiffness that allows them to wrap around large nerves while maintaining the oblong cross-sectional shape of the nerves, minimizing the distance between electrode contacts and neurons^47^. There is an extensive history of using C-FINEs to restore somatosensory feedback from the amputated hand^48^, and multiple recent studies have demonstrated that C-FINEs wrapped around the sciatic nerve and its branches can elicit tactile and proprioceptive sensations on the missing foot and leg in people with transtibial amputation^49^. These studies have shown that sensory restoration can improve balance^50^, gait kinetics, kinematics, and locomotor adaptation^51,52^. Epineural electrodes are thought to be more biocompatible than intraneural electrodes because they disrupt the blood-nerve barrier. Research on upper limb prosthetics has demonstrated that epineural electrodes, such as nerve cuffs and FINEs, can achieve a long-term (more than 6 months) stable interface with distal peripheral nerves^43,53^. In the case of lower limb amputation, stability and long-term performance have recently been characterized, with one study showing that nerve cuffs could reliably elicit sensory percepts of the lower limb amputees for up to 60 months and that reducing the stress on transition points in the electrode component improved the overall system performance^54^.

*Spinal cord stimulation (SCS)* is a common clinical procedure performed on approximately 50,000 people each year to treat various pain conditions^55^. Electrically stimulating the spinal cord can evoke sensations in the trunk and limbs^14,56^. Recent work has shown that lumbosacral SCS can evoke sensations in participants’ missing leg and foot according to dermatomal distributions of the spinal cord^14^. Participants reported a combination of naturalistic (e.g., sharp, vibration, pulsing, pressure, temperature, tickle) and paresthetic (e.g., tingle, buzz) descriptors for the evoked sensation. Additionally, in two people with transtibial amputation, using SCS for sensory restoration improved balance control and gait stability, and three participants experienced a clinically meaningful decrease in PLP when using SCS for sensory restoration^14^. Because the SCS leads sit outside the dura and have relatively large electrode contacts, they may be less sensitive to glial scarring than epineural or intraneural electrodes, though lead migration is a common clinical problem with SCS and could lead to instability of the evoked sensations^57,58^. In a recent study, anchoring the leads to subcutaneous fascia reduced lead migration, and sensations were stable over 90 days post-implantation in one participant^14^.

### Non-invasive somatosensory neuroprostheses

*Transcutaneous electrical nerve stimulation (TENS)* uses adhesive electrodes on the skin to activate the afferent nerve fibers beneath the skin’s surface. TENS can restore sensation in three ways: (1) by stimulating areas of skin re-innervated by nerves from the missing limb either naturally^59^ or via TSR^40,59^, (2) by stimulating proximal regions of superficial nerves to evoke sensation in the missing limb^60^, and (3) via sensory substitution (see below). Studies have shown that using TENS for sensory restoration can improve the perceptual embodiment of prosthetic limbs^61^, enhance gait kinematics^62–64^, reduce the metabolic cost of walking^65^, and decrease PLP^66,67^.

*Sensory substitution* involves stimulating alternative locations on the body to evoke sensations that do not appear to emanate from the missing limb but instead serve as a surrogate for those sensations. For example, technologies such as vibrotactile feedback use vibrotactors to deliver short, low-intensity vibrations to the surrogate body section. Vibrotactile feedback can be mapped to phases of the gait cycle^68^, helping to improve performance on functional tests such as the timed up-and-go test^69^ and reducing temporal step asymmetries^68^. Additionally, sensory substitution can enhance postural stability^70,71^. However, this method can increase cognitive effort, and substantial training is required to effectively integrate sensory information into prosthesis use^63^.

### Encoding of somatosensory information

Parameters such as stimulation amplitude, frequency, pulse width, and electrode location can be modulated to alter the intensity and location of the evoked sensation. For instance, sensors placed at the bottom of the prosthetic foot can be mapped to specific electrode contacts to evoke sensation at the same location in the missing foot. However, a limitation of current somatosensory neuroprostheses is that they often evoke non-natural sensations like buzz or tingling. Work to understand the optimal encoding parameters to elicit natural sensation is still in progress. Some of these approaches include linear, exponential, and biomimetic stimulation paradigms. During linear and exponential stimulation paradigms, amplitude^14^, pulse width^51^, or frequency^72^ is modulated to control the intensity of the perceived sensation, using either a linear or exponential^73^ transformation between sensor signals and stimulation parameters. With these paradigms, a stronger applied force maps to a larger value of the target stimulation parameter, resulting in a more intense sensation in the residual limb. Recent research has explored biomimetic stimulation, where frequency and amplitude are modulated simultaneously based on sensor signals and their derivatives to mimic the way populations of sensory neurons normally fire. This strategy can improve the naturalness of evoked sensations^44,74^, improve mobility, and reduce mental effort compared to traditional stimulation patterns^44^. For all of these strategies, a map must be built between the various stimulation parameters and the quality and intensity of perceived sensations. To quantify these maps, detailed psychophysical assessments such as two-alternative forced choice tasks can be used to objectively quantify the sensory experience during stimulation^14,21,36,59^.

## Prosthetic control

To improve users’ control of prosthetic devices, work has been done to develop technologies that decode neuromuscular signals from residual muscles to identify the user’s motor intent. This section will describe some of the recent developments in these myoelectric interfaces and the current methods used to decode neuromuscular signals for recognizing motor intent for lower-limb prosthetics.

### Myoelectric Interfaces

Electromyographic (EMG) signals are amplified neural control signals that activate muscles to produce body movement. Since the residual muscles that originally control the amputated limb are still innervated, EMG-based interfaces (also called myoelectric interfaces) are the most used neural interfaces for identifying user intent for prosthetic limb control^75,76^. EMG-controlled prosthetics enable amputees to directly control the prosthesis joint as if they were their natural limbs using model-based or machine-learning controllers^77^. They can also adjust the behavior of the prosthetic based on different muscle patterns to help the user cross different terrains^78^. Like somatosensory neuroprostheses, these myoelectric interfaces can be either invasive or non-invasive, with invasive procedures often leading to better signal quality and more neural information from the EMG recordings, while the non-invasive alternatives may be more widely accessible for those not wishing to undergo surgery.

#### Invasive myoelectric interfaces for prosthetic control

Invasive myoelectric interfaces have two advantages over non-invasive approaches. First, invasive surgical techniques can help access the signal when there is a high level of amputation, such that the muscles that control the distal joint are no longer available. For example, for transfemoral amputees, the muscles that control the ankle joint are gone. Thus, directly accessing the neural control signals from myoelectric interfaces for ankle control is impossible. Second, invasive technologies can record EMG signals from deep muscles inaccessible from EMG electrodes placed over the skin surface.

#### Surgical procedures

*Targeted Muscle Reinnervation (TMR)*, similar to TSR, is a surgical technique in which the nerves severed by amputation are rerouted to innervate the muscles that are biomechanically non-functional. TMR has been performed in patients with transtibial and transfemoral amputation and knee disarticulation procedures. For transtibial amputation, the major peripheral nerves (posterior tibial, deep and superficial peroneal nerves, saphenous nerve, and sural nerve) are sharply transected and transferred to motor nerve targets such as medial and lateral gastrocnemius, tibialis anterior and posterior, soleus, or peroneus longus or brevis^79^. For transfemoral amputation and knee disarticulation, the tibial and common peroneal nerves are transferred to motor branches of the gastrocnemius, semimembranosus, semitendinosus, and biceps femoris^80^. The reinnervated muscle amplifies the neural signals so that non-invasive EMG electrodes can record signals to control a prosthetic device, such as a lower-limb prosthesis^81^. In addition, this procedure has the added benefit of preventing neuroma and reducing PLP^82,83^.

*Regenerative Peripheral Nerve Interface (RPNI)* is a surgical procedure that uses an idea similar to TMR, except the reinnervation is performed on muscle grafts rather than residual muscles^84^. Over time, the muscle graft is reinnervated by the axons of the transected residual nerve, creating a bioamplifier for efferent motor action potentials, allowing for real-time prosthetic control^85^. This technique has been developed for above-knee amputations, below-knee amputations, and hip disarticulation procedures^84^. RPNIs have not been evaluated for prosthetic control for lower-limb amputees yet. However, this technique has been shown to be effective in reducing PLP and preventing the formation of neuromas in the residual lower limb^86^.

#### Implantable devices

*Invasive EMG (iEMG)* devices are implanted directly into the muscle tissue to record muscle activity. iEMG systems have been designed to transmit the EMG signal wirelessly to the prosthetic device without any transcutaneous leads^87^. They can be implanted directly into target muscles, including muscles that would be difficult to reach using surface EMG electrodes. While these interfaces have been used numerous times in upper-limb prosthetics, they are still relatively new to lower-limb prosthetics^75^.

#### Non-invasive neural interfaces for prosthetic control

*Surface Bipolar EMG (sEMG) electrodes* are placed on the skin surface over specific locations on the residual limb to record electrical activity from specific underlying muscles. A key design consideration for sEMG is how to interface these electrodes with the amputee’s socket, as the interface must be robust for stable EMG recordings during dynamic motions such as walking, without interfering with the socket’s fit or the user’s comfort. Several studies have used low-profile electrodes between the skin and socket or high-profile electrodes embedded in the prosthetic socket^88,89^. With advances in new materials and manufacturing technologies, flexible sensors^90^ or textile-based EMG sensors^91^ could be integrated into socks or gel liners for stable and reliable EMG recordings while maintaining user comfort^92^.

*High-density EMG (HD-EMG) interfaces* contain a high-density, evenly distributed grid of surface EMG electrodes. This interface is placed over a large area of residual limb without targeting specific muscles. The intent of these interfaces is either to record the activation patterns of surface muscles across the entire residual limb^93^ or to use EMG decomposition techniques to estimate motor unit action potentials, which contain more detailed efferent neural information than gross surface EMG signals^94^. Commercial flexible HD-EMG systems (such as the Quattrocentro system, OT Bioelettronica, Italy; Neurolife Technology, Battelle Memorial Institute, US) have been used in clinics and for upper limb prosthesis control in labs. Nevertheless, using HD EMGs for lower-limb prosthetic control has not yet been demonstrated.

### Decoding of motor intent and prosthesis control

**Fig. 2:**
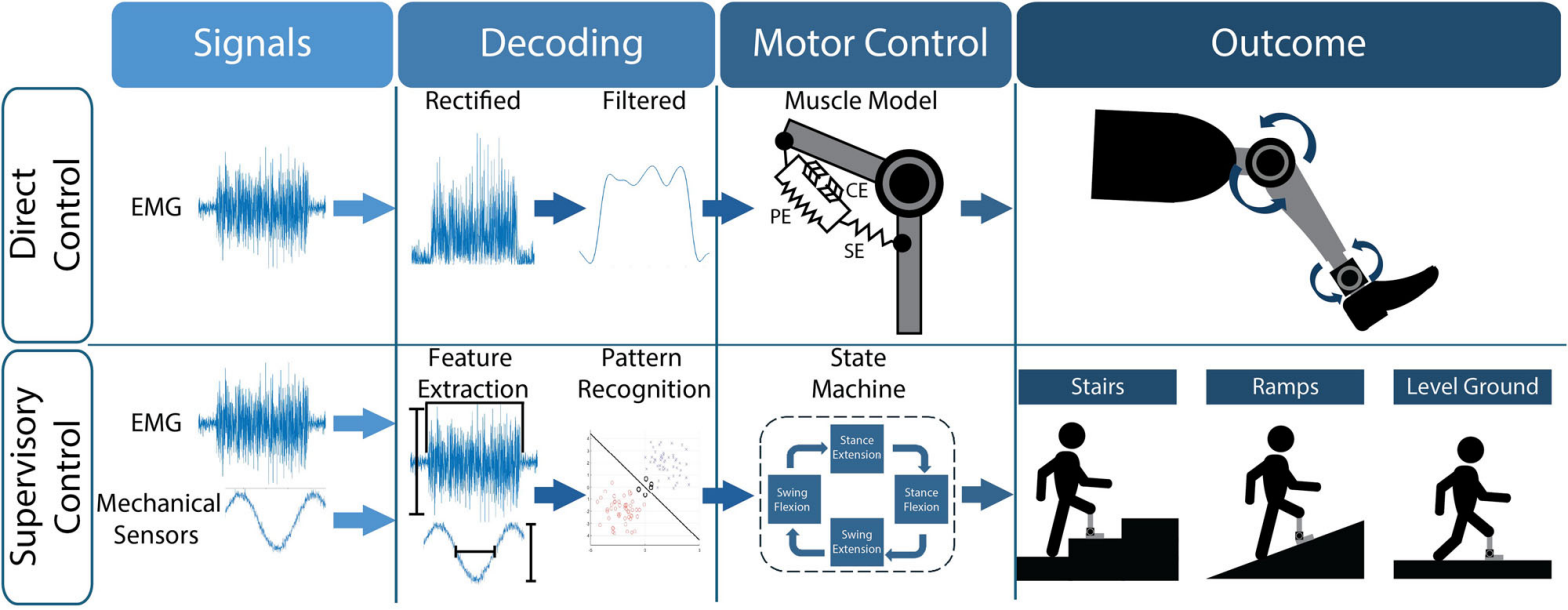
Block diagram of different EMG decoding strategies. Direct Control takes the recorded EMG signal, rectifies and passes that signal through a low-pass filter, then sends it through a computational muscle model such as a Hill-type muscle model, consisting of a contractile element (CE), parallel elastic (PE) element, and a series elastic (SE) element^95^. Finally, the model output drives either the torque or impedance of the prosthetic joints in a way that mimics natural muscle movements. Supervisory Control is a hybrid approach that uses both EMG signals and mechanical signals, such as ground reaction force or joint angle, to control the prosthetic device. In this case, features are extracted from the EMG and other sensor signals so that they can be run through pattern recognition algorithms to determine the motor intent of the user. These algorithms classify the user’s motor intent and alter the device’s state-machine controller to change the locomotive mode of the prosthetic device and allow the user to cross different types of terrain, such as stairs, ramps, or level ground.

Once the user’s EMG signals are recorded, a decoder is required to interpret the user’s motor intent to drive the prosthesis. Below, we describe the existing EMG decoding methods for lower-limb prosthetic control (Fig. 2).

*Direct Control in lower-limb prostheses* means that the output of the EMG decoder continuously and directly alters the mechanics of the prosthetic joints. EMG signals are first rectified and passed through a low-pass filter to transform them into smooth, continuous muscle excitation values. To approximate the user-intended control force or torque to be applied to the prosthetics’ actuators, the excitation values are used to activate Hill-type muscle models^95^ or pneumatic artificial muscle actuators^63^. Research groups have also applied linear combinations of excitation values between an agonist-antagonist muscle pair to estimate intended prosthetic joint control torque^96^ or impedance^97^. This approach has been tested on individuals with transtibial amputations in walking on uneven terrain^29^ and standing postural control^98^. A case study also showed that a person with a transfemoral amputation could use this approach to control a robotic knee prosthesis for walking and stair climbing^99^. The success of this approach relies on the user’s ability to coordinate antagonistic muscle pairs, which might not be possible for some amputations or for those with severe diabetic neuropathies.

*Supervisory control* uses myoelectric interfaces to select from different prosthetic locomotion control modes for different types of terrain. The interface only changes the mechanics of prosthesis joints during terrain transitions and is, therefore, discrete. The myoelectric interface in this case serves as a high-level supervisory controller; the low-level control still depends on an autonomous controller. EMG pattern recognition is used to identify the user’s intended locomotion mode, such as level-ground walking or stair ascent/descent. EMG features are extracted and sent to phase-dependent classifiers to determine the user’s locomotion intent for the next step^78^. Further studies have fused intrinsic mechanical sensor readings and EMG signals to improve locomotion mode recognition accuracy^81^. These studies showed that the method enables transfemoral amputees to negotiate changing terrains smoothly and seamlessly. However, pattern recognition may result in errors that can disrupt the walking stability of amputees. Additional research in designing a robust controller is necessary to make this myoelectric interface useful for daily use.

## Assessments of Advanced Sensory and Motor Prosthetics

Lower limb prosthetic users are at higher risk of falls^16^ and the use of the prosthetic requires a high degree of cognitive focus^18^. This review presents how different technologies improve participants’ functional outcomes. However, it is important to point out that many of the clinical assessments typically used for measuring balance and gait (e.g., functional gait assessment, 6 min walking test, sensory organization) are not sensitive enough to disentangle whether the higher risk of falls is due to missing sensory feedback^100^, lack of prosthetic control, or limitations of the prosthetic limbs. Developing better tests and outcome measures that can help us disentangle these deficiencies is an active area of research^101–106^. Recent studies have used innovative outcome measures such as a randomized ladder walking task^105^, walking on sand^107^, or obstacle avoidance during treadmill walking to assess balance and gait^52,108^. These tasks allow for quantification of the effects of restored sensory and motor control; however, further work must be done to improve their sensitivity and demonstrate validity and relation to fall risk.

## Future Directions

While multiple labs and some companies have focused on developing systems for restoring sensory feedback or motor control for upper-limb prosthetics, the development of similar approaches for lower-limb prosthetics is much more recent. Much can be learned from the efforts focused on upper-limb prosthetics, but as we described above, there are critical differences that must be considered for the lower limb (e.g., clinical challenges related to diabetes and increased risks associated with controller failure). Still, the clinical need for new solutions for lower-limb prosthetics is high, with the incidence of diabetic amputations increasing as the population ages. Fortunately, multiple cutting-edge neural engineering approaches that could have important impacts on lower-limb prosthetics are under development. These include extending surgical approaches such as RPNIs^86,109^ and AMIs^26^ to provide tactile sensory feedback, as well as new implantable technologies to record EMG signals from many muscles throughout the residual limb^110,111^.

However, despite increased research interest in both somatosensory restoration and myoelectric prosthesis control strategies for lower-limb amputees, the number of projects addressing closed-loop neural interfaces has been very limited. Sensorimotor integration is essential for human motor control and learning^112^. As such, we postulate that restoring the entire sensorimotor loop between a prosthetic limb and the human nervous system is essential to fully restore motor function for lower-limb amputees and to enable the embodiment of the prosthesis. We propose that neuroprosthetic development should focus on creating a fully integrated bidirectional system that provides somatosensory feedback, enabling users to gain proprioceptive information about both internal and external stimuli while enabling complete control of the prosthesis. While previous work has shown that somatosensory restoration alone can enhance outcomes such as overground walking speed and stair navigation^113^, combining sensory feedback with prosthetic control has been shown to improve these outcomes to a greater extent by allowing real-time modulation of the assistance provided by an active prosthetic limb^26,29^. A bidirectional system would enhance balance control, enable seamless transitions between tasks (e.g., from walking to stair climbing or navigating uneven terrain), and refine fine motor control of the prosthetic limb. However, developing such systems presents numerous open research questions that must be addressed. For example, we do not know whether high-fidelity control over sensory modality (e.g., touch, pressure, proprioception) is required or if paresthetic sensations (i.e., buzzing) will be sufficient to close the sensorimotor loop. Further, it is unclear what minimum somatosensory resolution is needed to effectively facilitate motor learning and improve the motor control of prosthetics in tasks related to gait and balance. Additionally, we do not know what lower-limb tasks can benefit most from a closed-loop neuroprosthesis, compared to the existing autonomous control or efferent EMG control only, nor do we have outcome measures of balance and gait that are likely to be sensitive to the effects of restored sensory feedback and improved motor control^100^. The knowledge gaps urge interdisciplinary collaborations among neuroscientists, roboticists, neural engineers, physical therapists, and physicians to improve the quality of life for individuals with lower-limb amputations.

## Data Availability

No datasets were generated or analysed during the current study.
